# Trophic and immunomodulatory effects of adipose tissue derived stem cells in a preclinical murine model of endometriosis

**DOI:** 10.1038/s41598-022-11891-5

**Published:** 2022-05-16

**Authors:** Toyofumi Hirakawa, Fusanori Yotsumoto, Naoto Shirasu, Chihiro Kiyoshima, Daichi Urushiyama, Kenichi Yoshikawa, Kohei Miyata, Masamitsu Kurakazu, Kaori Azuma Koga, Mikiko Aoki, Kazuki Nabeshima, Kaori S. Koga, Yutaka Osuga, Hiroaki Komatsu, Fuminori Taniguchi, Tasuku Harada, Shin’ichiro Yasunaga, Shingo Miyamoto

**Affiliations:** 1 Department of Obstetrics and Gynecology, Faculty of Medicine, Fukuoka University, 7-45-1 Nanakuma, Jonan-ku, Fukuoka, 814-0180 Japan; 2 Department of Biochemistry, Faculty of Medicine, Fukuoka University, 7-45-1 Nanakuma, Jonan-ku, Fukuoka, 814-0180 Japan; 3 Central Research Institute for Advanced Molecular Medicine, Fukuoka University, 7-45-1 Nanakuma, Jonan-ku, Fukuoka, 814-0180 Japan; 4 Department of Pathology, Fukuoka University, 7-45-1 Nanakuma, Jonan-ku, Fukuoka, 814-0180 Japan; 5 Department of Obstetrics and Gynecology, Faculty of Medicine, The University of Tokyo, 7-3-1 Hongo, Bunkyo-ku, Tokyo, 113-8654 Japan; 6 Department of Obstetrics and Gynecology, Faculty of Medicine, Tottori University, 36-1 Nishi-cho, Yonago, 683-8504 Japan

**Keywords:** Stem cells, Diseases

## Abstract

Endometriosis, which exhibits enigmatic pathological features such as stromal fibrosis and proliferation of ectopic epithelial cells, is known as a refractory disease. Mesenchymal stem cells modulate the fibrosis in stromal tissues through their trophic and immunomodulatory properties. To investigate the potential of stem cells in treating endometriosis, we examined the secondary morphology and molecular alterations in endometriosis-like lesions after the administration of adipose tissue-derived stem cells (ASCs) to an experimental murine model of endometriosis. The infused ASCs were found integrated in the endometriosis-like lesions. Accompanied by the suppression of stromal fibrosis and proliferation of endometriotic epithelial cells, the infusion of ASCs with stemness potential (early passage of ASCs) suppressed the growth of endometriosis-like lesions and inhibited the expression of pro-inflammatory and pro-fibrotic cytokines, whereas no significant attenuation of endometriosis-like lesions occurred after the infusion of ASCs without stemness potential (late passage of ASCs). Accordingly, the trophic and immunomodulatory properties of ASCs may regulate fibrosis in endometriosis-like lesions, suggesting that regenerative medicine could be recognized as an innovative treatment for patients with endometriosis through the accumulation of evidence of preclinical efficacy.

## Introduction

Endometriosis, a clinically common inflammatory gynecologic disorder, involves the growth of endometrial-like glands and stroma harboring reactive fibrosis and metaplasia outside the uterus^1^. It affects at least 6–10% of women with reproductive potential^2^ and is characterized by pelvic pain, dysmenorrhea, dyspareunia, infertility, dysuria, and dyschezia^3^. Moreover, ovarian endometriosis is found in 20–55% of women with endometriosis^4^, and accumulating evidence has demonstrated an association between ovarian endometriosis and ovarian cancer^5^. It has also been reported that malignant transformation occurs in up to 1% of patients with ovarian lesions^6^. Accordingly, women of reproductive age are prone to disorders associated with endometriosis. Endometriosis has been commonly treated by excising peritoneal lesions and ovarian cysts or administrating hormonal agents including progestin, oral contraceptives, and gonadotropin-releasing hormone agonists or antagonists. Such treatment strategies have been associated with high recurrence rates^7^; therefore, there is a need for more effective therapies for endometriosis.

The pathophysiology of endometriosis, which is characterized by fibrosis, resistance to apoptosis, and promotion of cell proliferation, has not yet been fully elucidated. Fibrosis is consistently found in all forms of endometriosis. Platelets, macrophages, ectopic endometrial cells, and sensory nerve fibers are involved in the development of fibrosis in all endometrial lesions^8^. Chronic inflammatory conditions have been linked to a variety of fibrotic disorders, including retroperitoneal fibrosis, hepatic fibrosis, idiopathic pulmonary fibrosis, systemic sclerosis (CREST syndrome), Crohn’s disease, radiation enteritis, collagenous colitis, encapsulating peritoneal sclerosis, and endometriosis^9^. Persistently high levels of TGF-β1 or stimulation by inflammatory cytokines (e.g., IL-6) induce adhesion and fibrosis in these diseases^10,11^. Thus, chronic exposure to pro-inflammatory and pro-fibrotic cytokines may inflict damage on indigenous MSC-like cells by triggering epigenetic modifications and activating matrix-related genes, resulting in myofibroblast differentiation and fibrosis^12^. According to these lines of evidence, it is plausible that the abnormal activation of TGF-β1 and upregulation of pro-inflammatory cytokines result in the development of fibrosis in endometriotic lesions^13,14^.

Stem cells possess self-renewal ability and can differentiate into a variety of specialized cell types, retaining infinite regenerative potential through trophic and immunomodulatory properties^15–19^. There are four main sources of stem cells: embryonic tissues, fetal tissues, adult tissues, and differentiated somatic cells^20^. In addition, genetically reprogrammed cells, referred to as induced pluripotent stem cells (iPSCs), can be obtained after the transfection of specific genes^20^. Regenerative therapy is defined as the therapeutic application of stem cells, progenitor cells, or both to repair damaged organs and restore their functions^21–24^. Thus far, embryonic stem cell- or iPSC-based therapies have not been successfully translated into clinical practice^25^. Meanwhile, mesenchymal stem cells (MSCs) are stromal cells harboring no regenerative properties but with trophic and immunomodulatory properties, including angiogenesis and immune modulation^25^. Regenerative therapies using such stem cells have been adapted in preclinical and clinical trials^21–24^. In principle, MCSs exert direct and indirect trophic as well as immunomodulatory properties to induce regenerative processes in damaged tissues^26^. MSCs directly exert trophic effects through the secretion of numerous paracrine factors via the secretosome and extracellular vesicles^26^. For its indirect effects, MSCs regulate the expression of pro-inflammatory and anti-inflammatory cytokines through the activity of T and B lymphocytes^26^. Thus, MSCs have been shown to be useful in treating a variety of chronic inflammatory disorders^27–33^.

To investigate the therapeutic potential of MSCs for the treatment of endometriosis, we generated an experimental murine model of endometriosis and subjected it to adipose tissue-derived stem cell (ASC) infusion. We then examined alterations in endometriosis-like lesions and assessed the accumulation of labeled ASCs in these lesions after treatment. In addition, we evaluated the gene expression of pro-inflammatory, anti-inflammatory, and pro-fibrotic cytokines as well as hormone receptors in the endometriosis-like lesions. Ki67 expression was also assessed to determine the proliferative index of endometriotic epithelial cells in endometriosis-like lesions after ASC treatment.

## Results

### Cellular characteristics and therapeutic effects of ASCs

**Figure 1:**
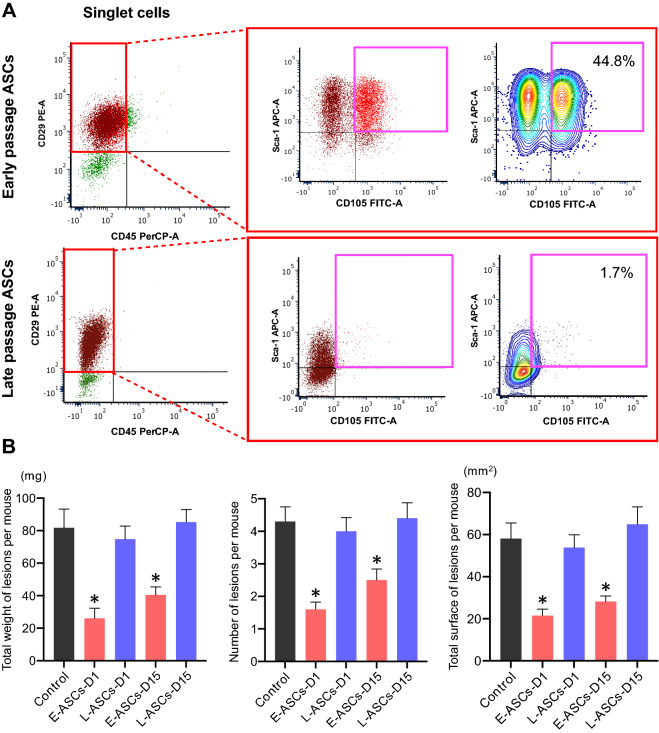
ASC stemness potential in endometriosis-like lesions. (**A**) Multicolor flow cytometry analysis of cells in the plot of singlet cells based on staining for CD105, CD29, Sca-1, and CD45. The percentages of mesenchymal stem cells, including early passage (3rd passage) adipose tissue-derived stem cells (ASCs), and late passage (20th passage) ASCs, harboring the cell-surface expression markers CD29, Sca-1, and CD105, and negative CD45 are shown. (**B**) Changes in secondary morphology of endometriosis-like lesion configuration after treatment with ASCs. *Control* intravenous administration of only PBS on day 1, *E-ASCs-D1* intravenous administration of early passage ASCs on day 1, *L-ASCs-D1* intravenous administration of late passage ASCs on day 1, *E-ASCs-D15* intravenous administration of early passage ASCs on day 15, *E-ASCs-D15* intravenous administration of late passage ASCs on day 15. Mice were sacrificed on day 28. n = 10 per group. Data are presented as the mean ± standard error. *P < 0.05 vs. Control.

To examine the expression of cell-surface markers of MSCs, flow cytometry was performed using antibodies against CD105, CD29, and Sca-1 as positive markers, and against CD45 as the negative marker. For cells in the P1, P2, and P3 plots, phenotype was analyzed based on stained cell-surface markers (Fig. 1A and Supplementary Fig. S2). The percentage of MSCs in ASCs was 44.8 ± 3.2% (n = 3) at the early cell passage (passage 3) (Fig. 1A), which decreased to 1.7 ± 0.3% (n = 3) by the late cell passage (passage 20) (Fig. 1A). These results suggest that serial subcultivation induces a marked decrease in the stemness properties of ASCs.

To investigate the anti-tumor effects of ASCs in the experimental models of endometriosis, we first assessed the total weight, number, and surface area of endometriosis-like lesions after the intravenous administration of phosphate buffer saline (PBS) as control (n = 10; Control), early passage of ASCs (n = 10; E-ASCs-D1), and late passage of ASCs (n = 10; L-ASCs-D1) to the experimental mice on day 1; the mice were then sacrificed on day 28. Next, to evaluate the anti-tumor effects of ASCs on the endometriosis-like lesions that had already formed, we estimated the total weight, number, and surface area of endometriosis-like lesions in mice with the intravenous administration of early passage ASCs (n = 10; E-ASCs-D15) and late passage of ASCs (n = 10; L-ASCs-D15) on day 15, which were then sacrificed on day 28. Meanwhile, the control mice (n = 10), treated with only PBS on day 1 and sacrificed on day 15, showed a total weight, number, and surface area of endometriosis-like lesions of 38.6 ± 4.7 mg, 2.5 ± 0.3, and 28.2 ± 2.5 mm^2^ (mean ± standard error), respectively. On day 15, mice in control had already formed the endometriosis-like lesions.

Endometriosis-like lesions grew in the abdominal cavities of all mice. These lesions were observed on the surface of the peritoneum, liver, kidney, intestinal membrane, and Douglas cavity. After the intravenous administration of ASCs on day 1 and day 15, the total weight, number, and surface area of the endometriosis-like lesions in E-ASCs-D1 and E-ASCs-D15 groups were significantly lower than those in the Control, L-ASCs-D1, and L-ASCs-D15 groups (Fig. 1B). In addition, no significant differences in the total weight, number, or surface area of endometriosis-like lesions were found between the E-ASCs-D1 and E-ASCs-D15 groups, among the Control, L-ASCs-D1, and L-ASCs-D15 groups (Fig. 1B). These results indicate that the administration of ASCs with stemness potential inhibits the progression of endometriosis-like lesions.

### Macroscopic and microscopic alterations in endometriosis-like lesions mediated by the administration of ASCs

**Figure 2:**
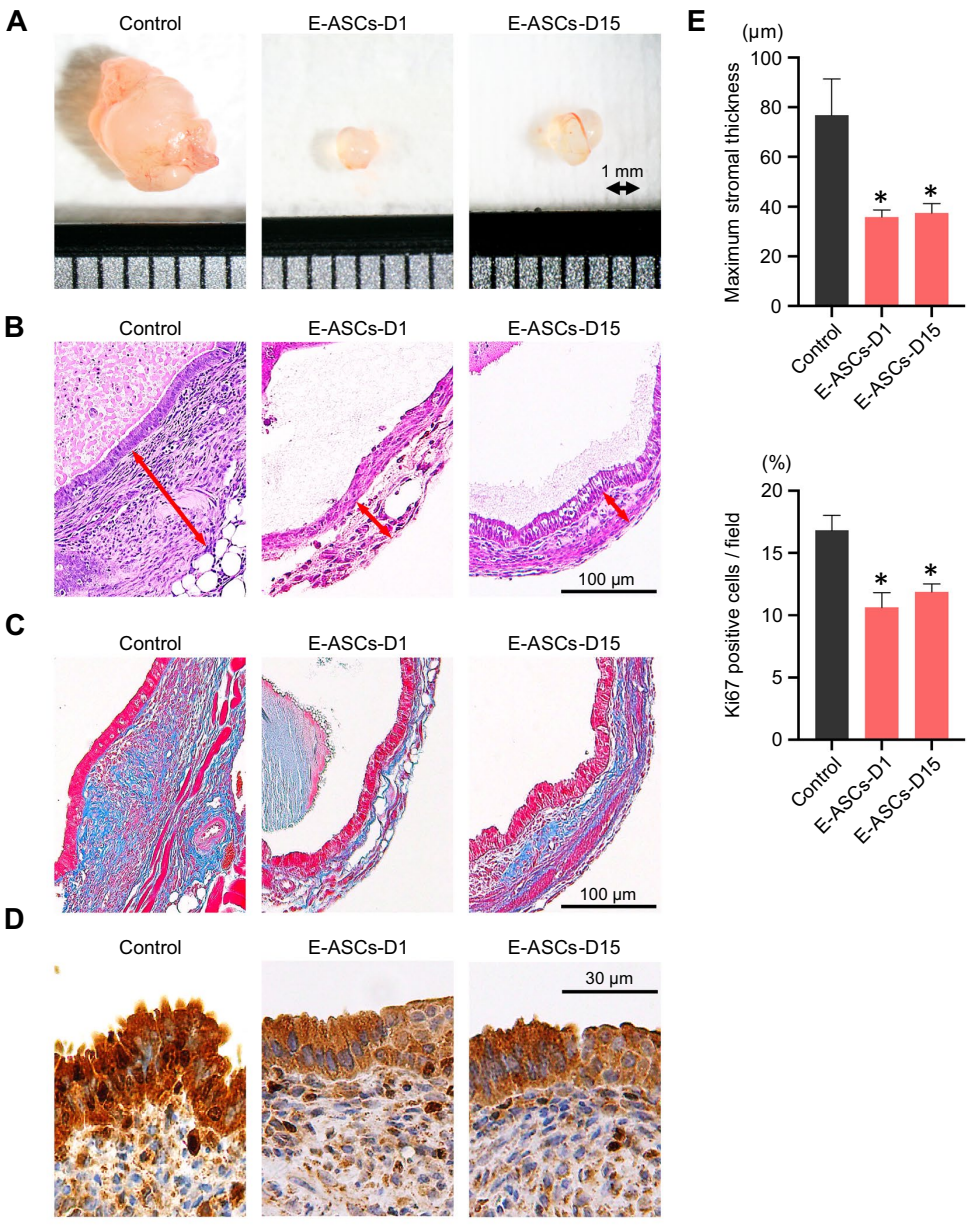
Macroscopic alterations and histopathology of endometriosis-like lesions after treatment with ASCs. (**A**) Representative excised implants from the endometriosis-like lesions. The interval at the bottom represents 1 mm. (**B**–**D**) Representative images of pathological specimens from excised implants stained with hematoxylin and eosin (**B**), Masson’s trichrome (**C**), and Ki67 (**D**). The red arrow in (**B**) indicates the maximum stromal thickness from the bottom of the endometrium to the surface of each cyst. The blue and red stains in (**C**) indicate collagen fibers and cells (mainly: fibroblasts) in the endometriosis-like lesions. (**E**) Quantification of maximum stromal thickness from the bottom of the endometrium to the surface of each cyst (top) and the percentage of Ki67-positive cells stained in the endometriotic epithelial cells (bottom). Data are presented as the mean ± standard error. *P < 0.01 vs. Control.

To evaluate the morphological effects of ASCs on endometriosis-like lesions, we examined the alterations in stromal tissues and endometriotic epithelial cells. Figure 2A shows a representative example of an excised implant from the Control, E-ASCs-D1, and E-ASCs-D15 groups. The endometriosis-like lesions formed simple cysts containing a clear fluid, and their diameter ranged from 1 to 10 mm. For evaluating stromal fibrosis, we measured the maximum stromal thickness from the bottom of the endometrium to the surface of each cyst after hematoxylin and eosin staining (Fig. 2B); most stromal tissues consisted of fibroblasts (stained in red) and collagen fibers (stained blue). The stromal tissues in endometriosis-like lesions were comprised primarily of collagen fibers (Fig. 2C). The maximum stromal thickness in E-ASCs-D1 and E-ASCs-D15 groups was significantly lower than that in the Control group (Fig. 2B,E). However, no significant difference in the maximum stromal thickness or stromal fibrosis was detected between E-ASCs-D1 and E-ASCs-D15 groups (Fig. 2B,C,E). These results suggest that endometriosis-like lesions gradually grow concurrent with stromal fibrosis and that infusion of ASCs with stemness potential inhibits the growth of endometriosis-like lesions and stromal fibrosis.

As for the endometriotic epithelial cells, we evaluated their expression of nuclear Ki67. The ratio of Ki67-positive cells in the E-ASCs-D1 and E-ASCs-D15 groups was significantly lower than that in the Control (Fig. 2D,E). These results suggest that endometriotic epithelial cells continue to proliferate in the endometriosis-like lesions, and that their proliferation is inhibited after infusion of ASCs with stemness potential.

### Alterations in target gene expression in endometriosis-like lesions mediated by the administration of ASCs

**Figure 3:**
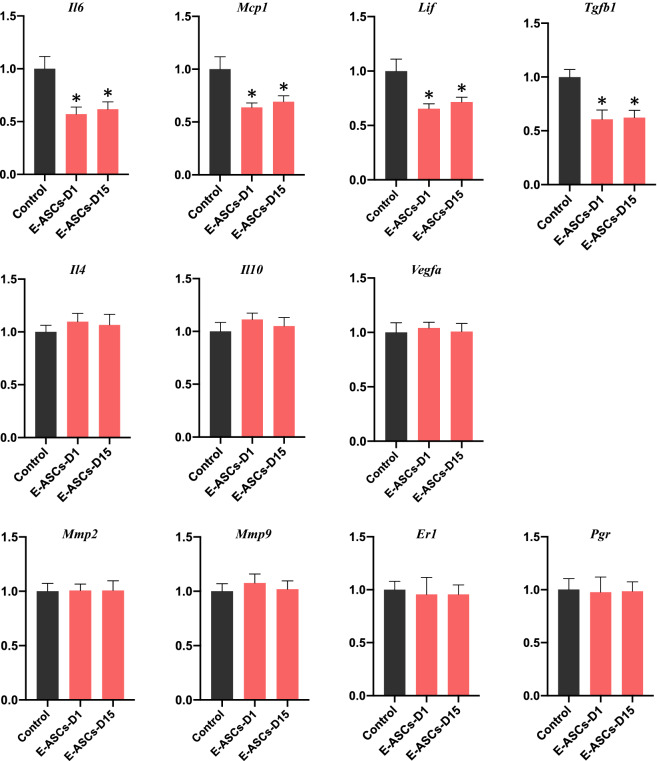
Changes in gene expression in endometriosis-like lesions after infusion of ASCs. Quantitative RT-PCR of pro-inflammatory (*Il6*, *Mcp1*, *Lif),* pro-fibrotic (*Tgfb1*), and anti-inflammatory cytokine (*Il4, Il10*) as well as angiogenetic factor (*Vegfa*), matrix metalloprotease (*Mmp2*, *Mmp9*), and hormone receptor (*Er1, Pgr*) gene expression levels in endometriosis-like lesions. Each group included 10 mice. Data are presented as the mean ± standard error. *P < 0.05 vs. Control.

To assess the molecular changes in endometriosis-like lesions induced by the administration of ASCs, we determined the mRNA expression levels of pro-and anti-inflammatory cytokines, pro-fibrotic cytokines, matrix metalloproteases, angiogenetic factors, and hormone receptors using quantitative RT-PCR. In the E-ASCs-D1 and E-ASCs-D15 groups, expression levels of monocyte chemotactic protein-1 (*Mcp1*), interleukin-6 (*Il6*), and leukemia inhibitory factor (*Lif*) (pro-inflammatory cytokines) as well as tumor growth factor-β1 (*Tgfb1*) (pro-fibrotic cytokine) were significantly lower than those in the Control group (Fig. 3). No significant differences in the expression levels of *Il10* and *Il4* (anti-inflammatory cytokines), matrix metalloprotease-2 (*Mmp2*) and *Mmp9* (matrix metalloproteases), vascular endothelial growth factor A (*Vegfa*) (angiogenetic factor), and estrogen receptor-1 (*Er1*) and progesterone receptor (*Pgr*) (hormone receptors) were found among the Control, E-ASCs-D1, and E-ASCs-D15 groups (Fig. 3). These results suggest that pro-inflammatory and pro-fibrotic cytokines are constantly expressed in endometriosis-like lesions, and that the administration of ASCs ameliorates endometriosis-like lesions by suppressing pro-inflammatory and pro-fibrotic cytokine expression.

### Tracking ASCs in endometriosis-like lesions in vivo

**Figure 4:**
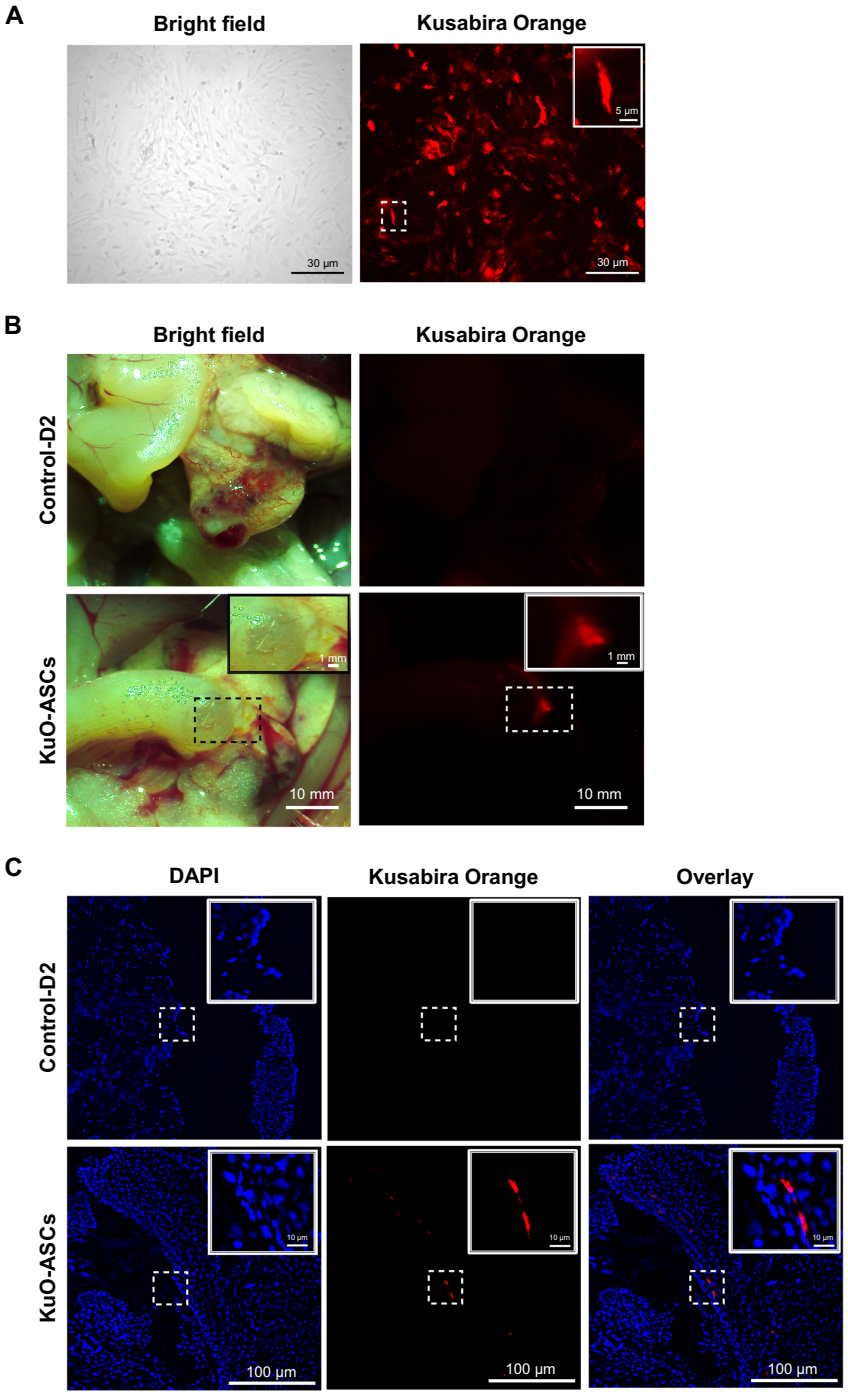
Localization of KuO-ASCs after intravenous administration. (**A**–**C**) Phase-contrast and fluorescence microscopy showing the cell morphology of Kusabira Orange (KuO)-expressing ASCs (**A**); macroscopic localization of KuO-ASCs in the endometriosis-like lesions (**B**); and microscopic localization of KuO-ASCs in the endometriosis-like lesions (**C**). Nuclei were counterstained with 4′,6-diamidino-2-phenylindole (DAPI).

In cell culture, Kusabira Orange (KuO)-ASCs originally displayed similar red/orange fluorescence as cells derived from KuO-expressing transgenic mice (Fig. 4A). Using phase-contrast and fluorescence microscopy, ASCs expressing KuO (KuO-ASCs), which were intravenously infused, appeared to accumulate in the endometriosis-like lesions (Fig. 4B). In the pathological specimens of endometriosis-like lesions, KuO-ASCs displayed spindle and small fibroblast-like cell morphologies, and fluorescence microscopy revealed that their cytoplasm expressed a strong red/orange fluorescent signal and their nuclei were strongly stained with 4′,6-diamidino-2-phenylindole (DAPI) (Fig. 4C). In addition, the intravenous administration of KuO-ASCs also revealed red/orange fluorescent signals in the pathological specimens of spleen and lungs (Supplementary Fig. S3). However, in other organs, including the brain, liver, kidney, heart, and peritoneum, no red/orange fluorescent signals were detected (Supplementary Fig. S3). These results suggest that intravenously infused ASCs predominantly accumulate in endometriosis-like lesions but may systemically spread and finally accumulate in the spleen or lungs.

## Discussion

In this study, the administration of ASCs significantly inhibited the growth of endometriosis-like lesions. In addition, ASCs displayed homing potential to integrate into endometriosis-like lesions and suppressed the proliferation of endometriotic epithelial cells as well as the interstitial stromal reaction in a preclinical murine model of endometriosis. ASC infusion also decreased pro-inflammatory and pro-fibrotic cytokine expression but did not alter the expression of anti-inflammatory cytokines, matrix metalloproteases, angiogenetic factors, or estrogen and progesterone receptors.

Endometrial stem cells, which are located in the basalis layer, differentiate into the endometrial stroma, epithelium, and endothelium^34^. After the infusion of bone-marrow cells with *mTert*-GFP or *Ch* β-*actin* GFP, all GFP-positive cells in the endometrium are immune cells, including T cells and macrophages, suggesting that bone-marrow stem cells do not contribute to endometrial cell lineages^35^. In this study, infused ASCs were primarily detected in the endometriosis-like lesions, suggesting that they help attenuate the stromal reaction in endometriosis-like lesions. Given that ASC administration significantly suppressed the thickness of the interstitial stroma and expression of pro-inflammatory and pro-fibrotic cytokines, it is plausible that ASCs, which were integrated into the institutional stromal tissues, suppress the expression of pro-inflammatory and pro-fibrotic cytokines that exacerbate the fibrotic changes in endometriosis. Indeed, ASC treatment significantly reduced the total number as well as the total weight and surface area of endometriosis-like lesions.

IL-6 levels are elevated in the peritoneal fluid, endometriotic lesions, and serum from women with endometriosis^36,37^, and TGF-β1 is also upregulated in the peritoneal fluid and peritoneal membranes of women with endometriosis^38,39^. In other preclinical models of endometriosis, it has been reported that the increase in IL-6 and TGF-β1 levels is attributable to the development of endometriosis-like lesions^13,14^. Elevated IL-6 and TGF-β1 levels at the inflammatory sites contribute to the recruitment of MSCs^40,41^. MSCs can reduce inflammation through the production of immunoregulatory molecules from MCSs as well as the accelerated switch from M1 to M2 macrophage, independently of their localization, for immunomodulatory effects^42,43^. Based on these evidence, MSCs, which retain immunomodulatory features and secrete trophic factors, have been utilized for treating various inflammatory and fibrotic diseases^44^. Several studies have demonstrated that MSC infusion attenuates fibrosis through the suppression of IL-6 or TGF-β1 expression^45,46^. In this study, the administration of ASCs also reduced the expression of IL-6, MCP-1, Lif-1, and TGF-β1, accompanied by the attenuation of endometriosis-like lesions. Although the immunomodulatory and trophic factors remain to be unidentified^47^, MCS infusion might have the potential of being a promising treatment strategy for endometriosis.

Although MSCs have been previously shown to suppress the function of anti-inflammatory and angiogenic cytokines^47^, such findings were not detected in this study. Here, the proliferation ratio of endometriotic epithelial cells in the treated groups was significantly suppressed and no clear differences in VEGF, MMP2, MMP9, IL-4, and IL-10 expression was found between the treated and control groups. In another experimental murine model of endometriosis, the infusion of anti-IL-6 antibody induced ectopic endometriotic epithelial cell atrophy^48^. Although it remains unclear why the growth of endometriotic epithelial cells was inhibited by ASC treatment, the suppression of IL-6 expression, which was mediated by ASC infusion, may have resulted in the decreased proliferation of endometriotic epithelial cells.

The safety and efficacy of infused MSCs have been validated by clinical trials in patients with a variety of diseases^40^. Moreover, ASC treatment had no influence on the expression of estrogen and progesterone receptors, suggesting that combination therapy involving hormonal therapy and MSC infusion would synergistically act to significantly attenuate endometriosis. Many women with endometriosis continue to suffer from severe dysmenorrhea, infertility, cancer, and other debilitating conditions. To better treat endometriosis, clinical trials are needed to fully evaluate the application potential of combined treatment with MSCs.

## Methods

### Animals

Six-week-old BALB/c female mice were obtained from KBT Oriental (Saga, Japan). A KuO-expressing transgenic C57BL/6 mouse was provided by Dr. H. Nakauchi at the Institute of Medical Science, University of Tokyo^49^. In the animal model of endometriosis, BALB/c female mice were used in both lesion donor and recipient. For treatment with ASCs against endometriosis-like lesions, the ASCs derived from BALB/c female mice were used in the experiments. In the homing experiment of ASCs, the ASCs derived from C57BL/6 mice, which expressed the fluorescent signals of Kusabira Orange, were used. All experiments were performed in accordance with the Guidelines for Animal Experiments at Fukuoka University and approved by the Animal Experiment Committee of Fukuoka University (approval number: 1806020, 2005007) and the Safety Committee for Recombinant DNA Experiments of Fukuoka University (approval number: 563). The study was carried out in compliance with the ARRIVE guidelines.

### Isolation of adipose-derived regenerative cells (ADRCs) and ASCs

We isolated ADRCs from the subcutaneous adipose tissues of BALB/c female mice and the KuO-expressing transgenic C57BL/6 mouse, as described previously^50^. ADRCs are almost identical to the stromal vascular fraction. To obtain ACSs, ADRCs were incubated on a 100 mm plate in 10 mL of MEMα/GlutaMax medium (Gibco, Grand Island, NY, USA) containing 20% fetal bovine serum (Gibco) and 1% antibiotic–antimycotic (Gibco) at 37 °C and 5% CO_2_ until 80% confluence. The medium was replaced every 3 days to remove nonadherent cells, and the adherent cells, which were considered ASCs, were passaged with TrypLE Express (Gibco). ASCs obtained at the early (P3: 3rd passage) and late passages (P20: 20th passage) were utilized, and cell numbers were counted using Luna-FL (Logos Biosystems, Anyang-si, South Korea). To determine the percentage of cells with stemness potential in ASCs of the early (P3: 3rd passage) and late passages (P20: 20th passage), the characteristics of ACSs were independently examined using flow cytometric analysis in triplicate.

### Animal experiments

The endometriosis murine model was established as previously described^51^ based on the retrograde shed endometrium theory. The experimental murine model of endometriosis is presented in Supplementary Fig. S1. Donor and recipient mice, which were ovariectomized after acclimation, were subcutaneously administered 100 μg/kg estradiol valerate (Tokyo Chemical Industry Co., Ltd, Tokyo, Japan) twice (day − 14 and − 7) for 2 weeks immediately after ovariectomy. Donor mice were sacrificed, and then their uterine horns were removed and minced as uterine fragment tissues. Half of the uterine fragment tissues obtained per mouse were suspended in 2 mL of PBS and injected into the peritoneal cavity of recipient mice with an 18-gauge needle (day 0). On day 28, these recipient mice were sacrificed, and the endometriosis-like lesions excised. Mice intravenously administered PBS (0.5 mL) on day 1, instead of receiving the ASCs infusion, were defined as control (Control group). Mice that were intravenously administered 1 × 10^6^ in the early passage of ASCs (P3: 3rd passage) and 1 × 10^6^ in the late passage of ASCs (P20: 20th passage) in PBS (0.5 mL) on day 1 and sacrificed on day 28 were defined as the E-ASCs-D1 and L-ASCs-D1 groups, respectively; before administration, the ASCs were suspended in 0.5 mL of PBS.

To confirm the anti-tumor effects of ASCs on endometriosis-like lesions that had already formed, recipient mice that had not received any treatment were sacrificed and endometriosis-like lesions were collected on day 15 after the injection of uterine fragment tissues on day 0. In addition, some mice were intravenously treated with 1 × 10^6^ in the early passage of ASCs (P3: 3rd passage) and 1 × 10^6^ in the late passage of ASCs (P20: 20th passage) in PBS (0.5 mL) on day 15 and sacrificed on day 28, after which endometriosis-like lesions were collected; these mice were defined as the E-ASCs-D15 and L-ASCs-D15 groups, respectively. For administration, 1 × 10^6^ ASCs in PBS or PBS only were injected into the tail vein of mice using a 25-gauge needle on day 1 or day 15.

### Flow cytometric, pathological, and immunohistochemical analyses

ASCs were suspended in a staining buffer (R&D Systems, Minneapolis, MN, USA), followed by incubation with a mouse primary antibody against the Fc component for 10 min at 4 °C to block nonspecific binding. They were then incubated for 30 min at 25 °C with CD105-FITC, CD29-PE, Sca-1-APC, and CD45-PerCP (all from the Mouse Mesenchymal Stem Cell [MSC] Multi-Color Flow Kit, cat. no. FMC003; R&D Systems). Flow cytometry was performed as described previously^50^. For histological staining, the collected tissues were formalin-fixed and then paraffin-embedded. Next, the tissue samples were sliced into 4-μm sections and stained with hematoxylin and eosin. Stromal thickness was evaluated by comparing the mean maximum width of each lesion. To evaluate fibrosis in endometriosis-like lesions, Masson’s trichrome staining was performed using pathological specimens of the lesions. To assess the proliferative ratio of endometriotic epithelial cells in endometriosis-like lesions, the primary antibody against Ki67 (1:200; Proteintech) was used. In a single section of each lesion, the Ki67-positive cell ratio was calculated by counting Ki67-positive epithelial cell nuclei per total epithelial cell nuclei. The pathological parameters, including the mean maximum width of the stromal tissues and proliferative ratio of endometriotic epithelial cells in endometriosis-like lesions, were independently estimated by three examiners (K.K., M.A., and K.N), and the mean value was defined as a representative value for each sample. Analyses of the pathological and immunohistochemical experiments were performed by different investigators blinded to the experimental animal groups.

### Quantitative reverse transcription PCR

Collected tissues were incubated in RNAlater reagent (Thermo Fisher Scientific, Waltham, MA, USA) at 4 °C overnight. Total RNA was extracted from tissues using the RiboPure RNA Purification Kit (Thermo Fisher Scientific), and the concentration and purity were estimated by measuring the A_260_/A_280_ ratio (where “A” denotes absorbance) on the NanoDrop 2000 spectrophotometer (Thermo Fisher Scientific). cDNA was generated by reverse transcription of the extracted RNA (1 μg) using the PrimeScript II 1st Strand cDNA Synthesis Kit (TaKaRa Bio Inc., Shiga, Japan). The mRNA levels were quantified using the StepOne real-time PCR system (Applied Biosystems, Waltham, MA, USA). TaqMan probes specific for *Mcp1*, *Il6*, *Lif*, *Tgfb1*, *Il4*, *Il10*, *Vegfa*, *Mmp2*, *Mmp9*, *Er1*, *Pgr*, and glyceraldehyde-3-phosphate dehydrogenase (*Gapdh*) were used for real-time PCR. The absolute values of *Mcp1*, *Il6*, *Lif, Tgfb1, Il4, Il10, Vegfa*, *Mmp2*, *Mmp9, Er1*, and *Pgr* were normalized to that of *Gapdh*, and the relative values were calculated by comparison with the control group. All samples were examined in triplicate.

### Tracking transplanted ASCs expressing KuO

KuO-ASCs were isolated from a KuO-expressing transgenic mouse. Approximately 1 × 10^6^ KuO-ASCs were injected either intraperitoneally or intravenously into the endometriosis model mice on day 1. To track the distribution of the infused KuO-ASCs in mice with endometriosis, a variety of organs, including the peritoneum, liver, spleen, kidneys, lungs, heart, and brain, were excised, and prepared as frozen sections at 24 h post-KuO-ASC infusion as previously described^52,53^. The tissue sections were fixed, washed, and incubated with DAPI. All sections were examined using a fluorescence microscope (Keyence, Osaka, Japan). Control mice were intravenously injected with 0.5 mL of PBS on day 1 and sacrificed on day 2 (Control-D2).

### Statistical analysis

All data were evaluated using the Prism 8 software (GraphPad Software Inc., San Diego, CA, USA). The Mann–Whitney *U* test and Benjamini–Hochberg method were used to compare the total weight, number, and surface area of the lesions; stromal thickness; percentage of Ki67-positive cells; and expression levels of target genes determined via quantitative RT-PCR among different groups. The data are presented as the mean ± standard error of the mean. *P* < 0.05 was considered statistically significant.

## Supplementary Information

Supplementary Information.

## Data Availability

The datasets generated during and/or analyzed during the current study are available from the corresponding author on reasonable request.
