# Supplementary material for: Trophic and immunomodulatory effects of adipose tissue derived stem cells in a preclinical murine model of endometriosis
Source: Sci Rep. 2022 May 16;12:8031. doi: 10.1038/s41598-022-11891-5 (PMC9110373; doi:10.1038/s41598-022-11891-5)
Supplement: Supplementary file 1 — Supplementary Information. [file 41598_2022_11891_MOESM1_ESM.docx]

**Supplementary Information**

**Trophic and Immunomodulatory Effects of Adipose Tissue Derived Stem Cells in a Preclinical Murine Model of Endometriosis.**

Toyofumi Hirakawa^1,2,3^, Fusanori Yotsumoto^1^, Naoto Shirasu^2^, Chihiro Kiyoshima^1,2,3^, Daichi Urushiyama^1^, Kenichi Yoshikawa^1^, Kohei Miyata^1^, Masamitsu Kurakazu^1^, Kaori Azuma Koga^4^, Mikiko Aoki^4^, Kazuki Nabeshima^4^, Kaori S Koga^5^, Yutaka Osuga^5^, Hiroaki Komatsu^6^, Fuminori Taniguchi^6^, Tasuku Harada^6^, Shin’ichiro Yasunaga^2^, and Shingo Miyamoto^1^*

^1^Department of Obstetrics & Gynecology, Fukuoka University, 7-45-1 Nanakuma, Jonan-ku, Fukuoka 814-0180, Japan.

^2^Department of Biochemistry, Faculty of Medicine, Fukuoka University, 7-45-1 Nanakuma, Jonan-ku, Fukuoka 814-0180, Japan.

^3^Central Research Institute for Advanced Molecular Medicine, Fukuoka University, 7-45-1 Nanakuma, Jonan-ku, Fukuoka 814-0180, Japan.

^4^Department of Pathology, Fukuoka University, 7-45-1 Nanakuma, Jonan-ku, Fukuoka 814-0180, Japan.

^5^Department of Obstetrics and Gynecology, Faculty of Medicine, The University of Tokyo, 7-3-1 Hongo, Bunkyo-ku, Tokyo 113-8654, Japan.

^6^Department of Obstetrics and Gynecology, Faculty of Medicine, Tottori University, 36-1 Nishi-cho, Yonago 683-8504, Japan.

***Corresponding author:** Shingo Miyamoto, MD, PhD

Director, Department of Obstetrics & Gynecology

Faculty of Medicine, Fukuoka University

7-45-1 Nanakuma, Jonan-ku, Fukuoka 814-0180, Japan.

Tel: +81-92-801-1011

Fax: +81-92-865-4114

E-mail: smiya@cis.fukuoka-u.ac.jp

**Supplementary Figures**

**Fig.S1. Schematic illustration of the experimental study design and confirmation of fibrotic tissues.** The upper panel shows the time schedule for establishing the experimental murine model of endometriosis, while in the lower panel, the time schedule, cell types, mediators, groups, and administration methods are presented. The arrows represent the time point for injections.

**Fig.S2. Distribution patterns of early (3^rd^ passage) and late (20^th^ passage) passages of ASCs, plotted as FSC-A (cell size) versus SSC-A (granularity), in the flow cytometric analysis.** The early (3^rd^) and late (20^th^) passages of adipose tissue-derived stem cells (ASCs) are identified as the cells in the P1 plot. Doublet exclusion was performed by plotting the heights or widths against the area for forward scatter (P2) or side scatter (P3) (FSC- and SSC-H/-W). The figure shows representative results. FSC-A: forward scatter area, SSC-A: side scatter area.

**Fig.S3. Localization of KuO-ASCs in various organs after intravenous administration.** Fluorescence micrographs showing localization of KuO-ASCs in the brain, liver, lungs, kidney, heart, spleen, and peritoneum after the intravenous administration of KuO-ASCs. Nuclei were counterstained with 4′,6-diamidino-2-phenylindole (DAPI). ASCs: adipose tissue-derived stem cells.
